# Thermochemical Transition in Non-Hydrogen-Bonded Polymers and Theory of Latent Decomposition

**DOI:** 10.3390/polym14235054

**Published:** 2022-11-22

**Authors:** Costas Tsioptsias

**Affiliations:** Laboratory of Physical Chemistry, Department of Chemical Engineering, Aristotle University of Thessaloniki, University Campus, 54124 Thessaloniki, Greece; ktsiopts@auth.gr

**Keywords:** simultaneous, decomposition, softening, latent, melting, glass transition, thermochemical

## Abstract

Although thermosets and various biopolymers cannot be softened without being decomposed, the vast majority of thermoplastics are believed to exhibit thermal transitions solely related to physical alterations of their structure—a behavior typical of low molecular weight substances. In this study, Differential Scanning Calorimetry (DSC), Fourier Transform Infrared Spectroscopy (FTIR) and Thermogravimetry (TGA) were used to study the softening of four common non-hydrogen-bonded thermoplastic polymers (polypropylene, polypropylene-grafted-maleic anhydride, poly(vinyl chloride) and polystyrene) along with a hydrogen-bonded polymer as a reference, namely, poly(vinyl alcohol). It is shown that the softening of these polymers is a thermochemical transition. Based on fundamental concepts of statistical thermodynamics, it is proposed that the thermal transition behavior of all kinds of polymers is qualitatively the same: polymers cannot be softened without being decomposed (in resemblance with their incapability to boil) and the only difference between the various types of polymers is quantitative and lies in the extent of decomposition during softening. Decomposition seems to reach a local maximum during softening; however, it is predicted that polymers constantly decompose even at room temperature and, by heating, (sensible) decomposition is not initiated but simply accelerated. The term “*latent decomposition*” is proposed to describe this concept.

## 1. Introduction

Material softening (melting/glass transition) and thermal decomposition are similar phenomena in the sense that a disturbance of bonds/interactions is involved in both cases. A primary difference is that in melting and glass transition, the physical interactions between molecules are loosened and no chemical bond rupture occurs, while in decomposition, the chemical bonds of molecules are broken. These phenomena, in the absence of suitable solvents—i.e., in the solid state—become mixed up in various polymers such as DNA, proteins, polysaccharides and thermosetting polymers, and an inherent relation between their thermal transition behaviors and the chemical changes of their structure can be identified. Such polymers are unable to exhibit actual melting or glass transition, that is, they cannot soften without decomposing. For example, the protein gelatin, upon heating, undergoes chemical changes before its thermal transition temperature is reached [1]. The polysaccharides cellulose and chitin (two very common and abundant organic substances) are other typical examples that do not exhibit any detectable thermal transition prior to decomposition. Some low molecular weight substances exhibit similar peculiarities, and during their decomposition some melting occurs. A specialized branch of kinetics, namely Bawn kinetics, has been developed to describe the influence of liquid phase formation during decomposition, i.e., how the decomposition rate is altered after fluidization [2]. Bawn kinetics has been mainly applied to pharmaceuticals and explosives [2], i.e., various such materials “melt” during decomposition, e.g., simultaneous melting and decomposition has recently been reported for some energetic materials [3]. A similar peculiarity in thermal behavior has been reported for lithium potassium tartrate [4]. Additionally, the variation in the various values for the melting point of succinic acid [5] in the literature, is suspected to have arisen from the decomposition/dehydration and formation of succinic anhydride upon heating. Interestingly, the vast majority of the above-mentioned polymers and low molecular weight substances, which exhibit an inability for actual melting/softening, are substances with increased capability for formation of hydrogen bonds. However, the vast majority of low molecular weight substances exhibit thermal transitions solely related to physical alterations of their structure.

This physical nature of thermal transitions has perhaps been taken for granted with regards to thermoplastic polymers. PVA is an exception to this, and simultaneous crystallization/melting and decomposition [6,7], or melting very close to decomposition [8], has been reported; however, melting and decomposition were treated as two independent effects. Recently [9], it was reported that the believed “melting” point of cellulose acetate butyrate (CAB), detected as endothermic peak in DSC, actually does not arise from melting but from decomposition which overlaps with the simultaneous softening of the material. It was pointed out that the co-occurrence of softening and decomposition is not accidental and should be treated as a unique effect. For this effect, the term “*glass chemical transition*” was initially proposed, which was then also seen to occur in cellulose acetate (CA) [10,11] and poly(vinyl alcohol) (PVA) [10,12]; the term “*thermochemical transition*” was proposed as more descriptive and general [10]. Moreover, it was reported that the believed “glass transition” of CAB is also a thermochemical transition [9]. Recently the concept of consecutive heating cycles [6,7] was adopted to re-examine the thermal behavior of PVA with respect to the thermochemical transition [12]. It was concluded that the glass transition of PVA is a thermochemical transition with no detectable mass loss. This interpretation could provide an explanation for the various contradictions regarding the existence of, or extent of, decomposition derived from different analytical methods, e.g., signs of decomposition could be detected in the FTIR results, but no mass loss was detected in TGA [12]. Additionally, it was shown that the specific heat of “melting” measured by DSC is governed by the heat required for decomposition, which can be expressed in an alternative way to heat of fusion (to take into account the decomposed mass rather than the overall mass of the sample). It was also discussed that the experimental values of the specific heat of thermochemical transition may be characterized by large uncertainty due to various factors. One major factor is the fact that some portions of the decomposition products may not be able to vaporize [13] at the temperature range of the thermochemical transition and thus, the heat measured by DSC does not correspond to the mass loss detected by TGA [12]. In addition, recently the latent limit of detection (LLoD) of TGA was reported [14]. Briefly, it was reported that due to the buoyancy exerted on the sample during a TGA measurement, there is a hidden limit (LLoD) for the % degree of actual mass loss which must be surpassed in order for the TGA sensor to be able to detect (apparent) weight loss [14]. Thus, the combination of the absence of volatility of the polymers’ decomposition products in the early decomposition stage, along with LLoD of TGA, can provide a reasonable justification for the occurrence of decomposition with no detectable mass loss in TGA, as is the case for the glass transition of PVA [12].

Almost simultaneously with the above mentioned studies regarding polymers, the thermochemical transition was also reported to occur in some low molecular weight substances, namely two flavonoids (silybin [15] and quercetin [16]) and gallic acid [17]. It was reported [16] that the thermochemical transition is the “connection” among three different aspects [16]. These three aspects are: (1) the inconsistencies detectable in the literature regarding the experimental values of the thermal thermodynamic properties of such (pharmaceutical) substances [15,16,17]; (2) the reported difficulty and poor efficiency of predicting the melting point of pharmaceuticals [18,19]; (3) the fact that Bawn kinetics has been applied to study pharmaceuticals [2].

In these recent studies of thermochemical transition, all of the substances (both polymers and low molecular weight substances) comprised molecules with increased hydrogen bond formation. It was recognized that the increased hydrogen bonding formation favors thermochemical transition through a double effect [10,11,15,16,17]: (1) hydrogen bonding hinders melting by keeping molecules close to each other by strong interaction and (2) it facilitates decomposition through the weakening of chemical bonds. For the O-H chemical bond, by FTIR spectroscopy it was found that in such substances, the free and strongly bounded hydroxyl groups may vary up to ~30% in their chemical bond strength [11,15,16]. This weakening of chemical bonds enables decomposition reactions, and for cellulose esters [9,10,11] and gallic acid [17] it was reported that the primary decomposition reaction is dehydration caused by esterification.

In general, the decomposition of polymers is a complex effect which constantly attracts research interest, e.g., recently, the enhanced char formation during thermal decomposition of poly(vinyl chloride) (PVC) was studied [20], as well as the decomposition mechanism of various vinyl polymers [21]. However, decomposition of polymers does not seem to be fully understood (especially the decomposition at low temperatures); recently, it has been reported that the use of high temperature data for modelling decomposition at lower temperatures resulted in poor agreement between predicted and real data and a new equation was proposed in order to take into account the induction period of the decomposition process at low temperatures [22]. Similarly, the recent finding of thermochemical transition in some thermoplastic polymers points out that the thermal behavior of polymers may need further understanding. Of course, thermal analysis in general is continuously developed and updated, e.g., see [23]. However, among other effects, the four following effects in the thermal transition behavior of polymers, to the best of the author’s knowledge, are either under debate or not at all discussed: (1) the typical broad range/asymmetry of the melting peaks of polymers, (2) the small endothermic peak in the glass transition temperature range, (3) the existence of melting in both signals (reversing and non-reversing) of modulated DSC and (4) the decrease in the heat capacity just after melting which can be seen in the DSC curves of various polymers in the current body of literature. Interpretations of these four effects are briefly discussed below.

The typical broad range/asymmetry of the melting peaks of polymers is commonly attributed to successive melting of crystallites of different sizes [24]. Endothermic peaks, which are sometimes observed at the end of the glass transition of polymers, are interpreted solely in physical terms and, specifically, are attributed either to enthalpy recovery [25] or related to melting [26]. Endothermic peaks in the melting temperature range, which are detected in the non-reversing signal of modulated DSC, e.g., isotactic poly(propylene) (PP) [27] and poly(L-lactic acid) (PLLA) [28], are attributed to the melting of crystals formed by recrystallization of another form. In general, it is accepted that “most melting” is detected in the reversing signal and “some melting” in the non-reversing signal of modulated DSC [29], [30]. In other words, it seems that some polymer crystals behave differently to others in the modulation of temperature. To the best of the author’s knowledge, there is no available explanation for this. Recently, for CAB [9] it was proven that the endothermic peak detected in the non-reversing signal of modulated DSC (and in the signal of the conventional DSC) is related to decomposition (thermochemical transition) and not to melting.

There is another observation (the above mentioned fourth major effect) related to the melting peak of polymers as detected in DSC which is worthy of discussion. Even for low molecular weight substances, no general statement can be made about the alteration of density and of the (specific) heat capacity during melting [31] and, usually but not always, small changes in (specific) heat capacity occur [31]. The overall change in (specific) heat capacity during melting is the result of various effects, some of which tend to increase (specific) heat capacity and others tend to decrease it [31]. For most polymers, the (specific) heat capacity in the liquid state is higher than that of the solid state [31,32]. However, for most polymers in a simple DSC scan, the baseline (or in other words the DSC signal which is directly related to the heat capacity of the polymer) just after the “melting” peak is decreased; that is, it is less endothermic compared to its values before the occurrence of “melting” (see Figure S1 and related text in supporting information). This suggests that the heat capacity of the sample has decreased during “melting”. This effect can be detected in the DSC curves of various polymers such as (suggestively): isotactic PP [33,34], poly(ethylene terephthalate) (PET) [35] and poly(l-lactic acid) PLLA [36]. To the best of the author’s knowledge, there is no available explanation for this effect.

Independently of the partial or full correctness of the available interpretations for these effects, it is clear that the correctness of an interpretation cannot guarantee its exclusiveness. The scope of this study is to demonstrate that the thermal transitions (melting or glass transition) of polymers are not purely physical and occur simultaneously with decomposition, and that this behavior is an inherent property of all polymers.

## 2. Materials and Methods

Isotactic PP with an average Mw of ~340,000 g/mol and an average Mn of ~97,000 g/mol was purchased from Sigma-Aldrich. Poly(styrene) (PS) (BDH limited) with Mw of ~100,000 g/mol was used. PVA (Aldrich) 99+% hydrolyzed with Mw in the range 89,000–98,000 g/mol was used. PVC (Aldrich) of very high molecular weight was used. Polypropylene-g-maleic anhydride (PP-g-MA) (BONDYRAM^®^ 1001) with a melt flow index (190 °C, 2.16 kg) of 100 g/10 min and 1% maleic anhydride (MA) content was also used. KBr (Chem-Lab) of purity 99.5+% was used for the FTIR measurements. Indium was used as the standard sample for the DSC (see Section 1 of Supporting Information for more details).

A Shimadzu DSC-50 differential scanning calorimeter, a Shimadzu TGA-50 Thermogravimetric Analyzer (TGA), a Biorad FTS-175 Fourier Transform Infrared spectrometer (FTIR) and a Sartorius scale (model B 120S, ±0.0001 g) were used.

DSC and TGA measurements were performed under nitrogen atmosphere (flow 20 mL/min) and a heating rate of 10 °C/min. Empty pan measurements were also performed in DSC and TGA in order to take into account instrument drift. The DSC and TGA raw data were corrected by subtraction of the empty pan measurement (see Sections 1 and 2 of Supporting Information for more details). For all samples, three consecutive heating cycles were performed. The cooling between the heating cycles was not controlled, and it occurred slowly at room temperature (the pans were kept under nitrogen flow during cooling and were not exposed to room air). In the DSC, PP and PP-g-MA were heated from 40 to 190 °C, PS from 40 to 130 °C, PVC from 40 to 180 °C and PVA from 40 to 250 °C. In TGA, PS, PP and PVA were heated at the same temperatures as in DSC while PP-g-MA was heated from 40 to 180 °C and PVC from 40 to 250 °C. For PP, additional DSC and TGA measurements were performed up to 350 °C. Details about the concept for selecting these temperature ranges can be found in Section 3 of the Supporting Information. It should be mentioned that a total of 16 TGA measurements were carried out (3 for every polymer plus one additional for PP up to 350 °C). However, only 7 measurements will be presented and discussed, specifically the ones in which the mass loss is indisputably detected in the raw data.

Each polymer (except PVA) was mixed with KBr (at polymer-to-KBr-mass ratio ~1:200) and processed into pellets (hydraulic press, 100 Bar). These pellets were measured by FTIR. Then, the KBr pellets (along with the KBr reference pellet) were first placed in a glass reactor and purged with nitrogen gas. They were then heated inside an air oven from room temperature up to 170 °C for 5 min, and after cooling (at room temperature), the pellets were measured again with FTIR. This process was repeated another two times. All samples were heated up to 170 °C, except for PS which was heated up to 130 °C (three times in total). All FTIR measurements were performed by collecting 64 scans with a resolution of 2 cm^−1^ in absorbance mode. For each polymer, the raw spectrum of the raw material is presented, while the spectra of the samples after each heating are presented as subtracted spectra (e.g., 2nd–1st heating). Prior to spectra subtraction, baseline correction was applied. Wherever needed, spectra multiplication by an appropriate factor was performed to increase signal-to-noise ratio. Details about the concept of the FTIR measurements and the adopted approach can be found in Section 4 of the Supporting Information.

## 3. Results and Discussion

To enable the reader’s understanding, a short description of the structure of the article will now be presented. Five different polymers (PVA, PP-g-MA, PP, PVC and PS) were studied under three consecutive heating cycles, by DSC, TGA and FTIR. Firstly, the results for PVA will be presented and interpreted. PVA was recently studied [12] under four consecutive heating cycles and thus no FTIR results will be presented; however, further insights on the thermal behavior of PVA will be provided that focus on the (apparent) heat capacity values before and after thermochemical transition. Then, the results for other polymers will be presented, and it is shown that the same interpretations as those made for PVA can be applied to other polymers which exhibit no capability for hydrogen-bond formation (PP-g-MA is an exception, and the cause of this is critically discussed). The discussions and the interpretations of the results for all polymers are mainly focused on three aspects: (1) the decrease in the heat capacity at the end of melting or glass transition (see Introduction section), (2) the inconsistencies among TGA and DSC regarding the initiation of decomposition and, more precisely, the initiation of mass loss in TGA without detecting, in the same temperature range, the heat required for decomposition in DSC and (3) the presence of impurities and the low probability of them being exclusively responsible for the observed behavior. However, a brief discussion of the effect of impurities is also included. Finally, based on fundamental concepts of statistical thermodynamics, theoretical support and explanations for the experimental observations and interpretations will be presented and, specifically, a theory will be proposed which describes the thermal behavior of all kinds of polymers under a unified basis.

Before proceeding, it is worth mentioning that the term “first heating” is used throughout the text for describing the sequence of the experimental measurement, and not in the sense that the polymer raw material is heated for the first time ever. Raw polymers may have been already heated during their production. Of course, even if all of the particular polymer samples had already been heated during their production, none of the conclusions of this study are affected.

### 3.1. PVA

**Figure 1:**
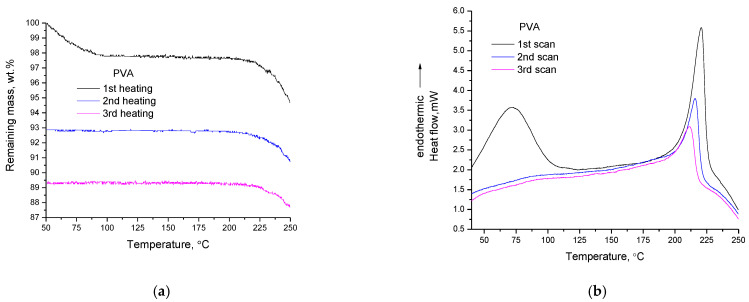
(**a**) Three consecutive TGA heating cycles of PVA; (**b**) Three consecutive DSC scans of PVA.

Three consecutive TGA measurements of PVA are presented in Figure 1a and the respective DSC curves are presented in Figure 1b. From the TGA curves it is apparent that in every subsequent heating cycle, the mass loss in the temperature range of the thermochemical transition (around 200–230 °C) is decreased. From the DSC curves it is apparent that in every subsequent cycle, the heat of “melting” is decreased. These have been recently interpreted. Briefly, it was confirmed by FTIR spectroscopy, stereoscopy and macroscopic observations (black color), that due to decomposition, regions of increased thermal stability are formed and thus, in the next heating cycle, during thermochemical transition, fewer regions are susceptible to decomposition (lower mass loss in TGA) [12]. Consequently, less heat is required/absorbed in the next cycle (less heat is detected in DSC). In addition, it was reported that the glass transition of PVA is a thermochemical transition with no detectable (in TGA) mass loss.

In the previous study regarding PVA [12], the heat of decomposition was examined along with the mass loss, but there was no discussion about the alterations of the heat capacity during thermochemical transition. In general, the DSC signal (*s*) is influenced by the heat absorbed by the sample (apparent heat capacity). This heat equals to the heat capacity (*c*) of the sample that is, mass (*m*) times specific heat capacity (*C_p_*) plus any heat (*Q*) that may be absorbed or released during heating, e.g., heat for vaporization of impurities, heat for melting, decomposition, etc. That is, *s = c + Q = m × C_p_ + Q*.

From the DSC curves it is obvious that, independently of the number of heating cycles, besides the overall asymmetry of the peak, the DSC signal of PVA is decreased after the thermochemical transition (end of the peak) compared to the respective value at the beginning of the transition (also see Introduction and Figure S1 in Supporting Information). In addition, the DSC signal after the endothermic peak is constantly decreasing (shifting towards exothermic direction). This clearly points out that the heat absorbed by the sample (DSC signal, s) is constantly decreasing. The following four factors can potentially contribute to this decrease:

(1) Decrease in s due to decrease in *c* (*=m × C_p_*) due to decreased *C_p_* values of decomposition products. More specifically, due to decomposition, a portion of the polymer mass transits to the vapor phase in the form of volatile decomposition products. Such products may have decreased specific heat capacities compared to that of the polymer. Thus, this can cause the DSC signal to shift towards the exothermic direction.
(2) Decrease in s due to decrease in *c* (*=m × C_p_*) due to decrease in mass (*m*) of the sample. The DSC pans used in this study did not seal hermetically. If increased vapor phase is formed, then a leak may occur and thus, an actual mass loss out of the DSC pan can occur. This, again, can cause the DSC signal to shift towards the exothermic direction.
(3) Decrease in s due to decrease in *c* (*=m × C_p_*) due to the decrease in the *C_p_* of PVA. More specifically, the decomposed (residue) regions formed in PVA [12] are expected to be characterized by decreased thermal mobility and decreased number of available conformations and these are translated to decreased *C_p_* values. This can cause the DSC signal to shift towards exothermic direction. It is stressed that the alteration of the specific heat capacity of polymers caused by decomposition is not unambiguous and competitive effects are involved. Decomposition, on the one hand, tends to decrease the specific heat capacity due to new stable regions which are formed due to decomposition and are characterized by constrained conformations; however, on the other hand, decomposition tends to increase the specific heat capacity by increasing free volume and the possible conformations. The number and size of the pendant groups and the decomposition pathway (random scission, etc.) are some of the factors that are involved in determining which of the two trends will ultimately prevail for a given polymer and at a given temperature.
(4) Decrease in s due to appearance of exothermic *Q*. Especially in the early stages of decomposition through the random scission pathway, large decomposition fragments are formed which are incapable of vaporizing [13]. These large fragments initially have highly reactive sites, e.g., free radicals, and react with each other (or with the polymer residue). The formation of new chemical bonds releases energy. This can cause the DSC signal to shift towards the exothermic direction.

It should be mentioned that the above four effects are not contradictive to each other and can simultaneously contribute, at different extents, to the exothermic shift of the DSC signal. From the above, it can be concluded that the decrease in the apparent heat capacity of PVA during and after the thermochemical transition is related to decomposition. If just melting occurred, then the mass would be constant and thus the DSC signal after the peak should be higher than its value at the beginning of the peak to reflect the increase in *C_p_* by increasing temperature. The decrease in the heat capacity during the thermal transition will be mentioned throughout the text when discussing/interpreting the thermal behavior of other polymers.

**Figure 2:**
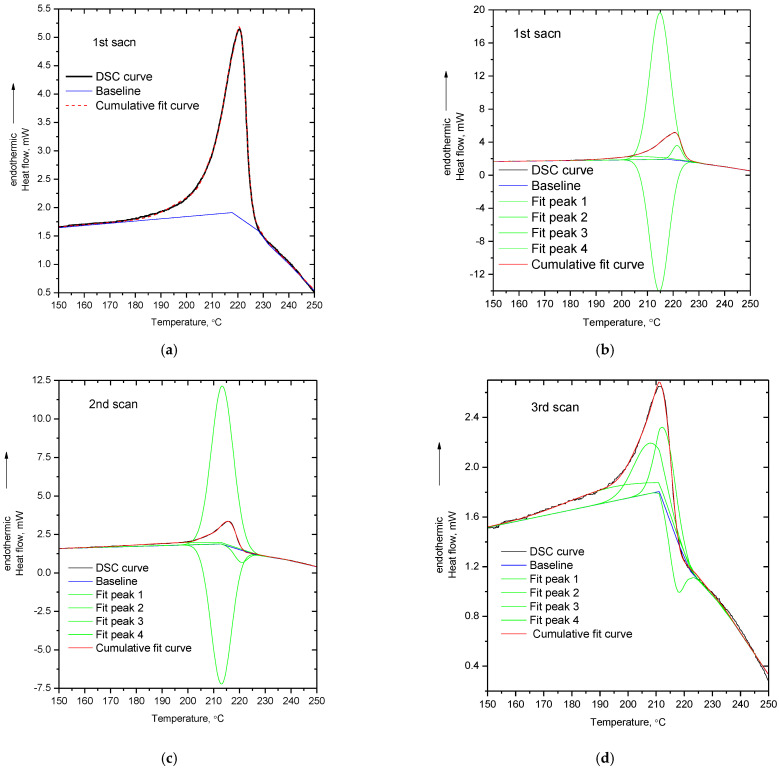
(**a**) DSC curve (first scan) of PVA, cumulative fit peak and baseline used for fitting; (**b**) Fitting of the DSC curve (first scan) of PVA; (**c**) Fitting of the DSC curve (second scan) of PVA; (**d**) Fitting of the DSC curve (third scan) of PVA.

In order to provide more insights for the DSC signal alterations, curve fitting (with four Gauss peaks) was performed on the DSC peaks presented in Figure 1. As it has already been pointed out [12], the determination of the proper baseline for integrating or performing fitting in a thermochemical transition peak is complex, since the baseline is altered during the transition due to the various competitive and overlapping effects. In Figure 2a, the DSC curve from the first scan of PVA, along with the cumulative fit peak and the baseline, are presented. As can be seen, in order to take into account the fact that mass loss occurs during the thermochemical transition, a baseline comprising two linear parts was adopted. The two linear parts of the baseline intersect at the maximum of the DSC peak. The first part of the baseline is characterized by the endothermic slope, representing the expected increase in heat capacity caused by an increase in specific heat capacity, due to an increase in temperature, or heat absorption due to decomposition. The second part of the baseline has an exothermic slope, representing the decrease in the heat capacity caused either by the decrease in mass, due to decomposition or/and the heat release from the exothermic reactions among the decomposition products. In Figure 2b–d, the fitted peaks for the three DSC peaks of the respective scans of PVA are presented. As can be seen, in all three scans, at least one negative peak is needed for fitting the DSC curve. It should be mentioned that in the preliminary fitting trials, it was observed that no satisfying coincidence of the raw and cumulative fit peak was achieved if fitting was performed with two or three fit peaks instead of four. The necessity for performing fitting with four peaks arises from the high asymmetry of the DSC peak of PVA. Additionally, it should be noted that the position as well as the areas of the fit peaks are baseline sensitive. For example, by altering the baseline, four positive peaks, or more than two negative peaks, could be obtained; however, above, the reason for choosing this baseline was justified. It is stressed that after the determination of the baseline, the fitting procedure was performed without setting any requirements/conditions, e.g., at least one of the fit peaks being negative. As mentioned above, in order to describe the overall peak, at least one negative peak is needed.

The physical meaningfulness of the negative peak is related to the above mentioned forth factor which can potentially contribute to the decreased heat capacity values at the end of the melting peak. Specifically, some of the decomposition fragments (especially in the early stage of decomposition) are expected to be too large to vaporize [13]. The fragments, initially, are not inert molecules but free radicals and thus they are highly reactive. These reactive intermediate products will react either with each other or with the polymer residue, and new covalent bonds will be formed. Since the breakage of such bonds is endothermic, the formation of new bonds will be exothermic, that is, heat will be released. These exothermic reactions occur simultaneously with the endothermic decomposition reactions which are responsible for the production of the intermediate decomposition products. Thus, the negative fit peak, as well as the large endothermic fit peak (much larger than the DSC peak), express the overlapping of the endothermic decomposition reactions and exothermic reactions among the decomposition products.

To summarize the discussions of this section, it seems that the high asymmetry of the DSC peak as well as the decrease in the DSC signal at the end of the endothermic peak can be interpreted and understood in terms of decomposition. Decomposition causes exothermic shifts of the DSC signal due to mass loss (decrease in mass inside the DSC pan) and due to exothermic heat released by reactions among the decomposition products (this contribution is expected to be more intense if the decomposition products are large fragments and consequently are not volatile). The overlapping of such exothermic shifts with the heat absorption required for further decomposition leads to the asymmetry of the DSC peak.

### 3.2. PP-g-MA

**Figure 3:**
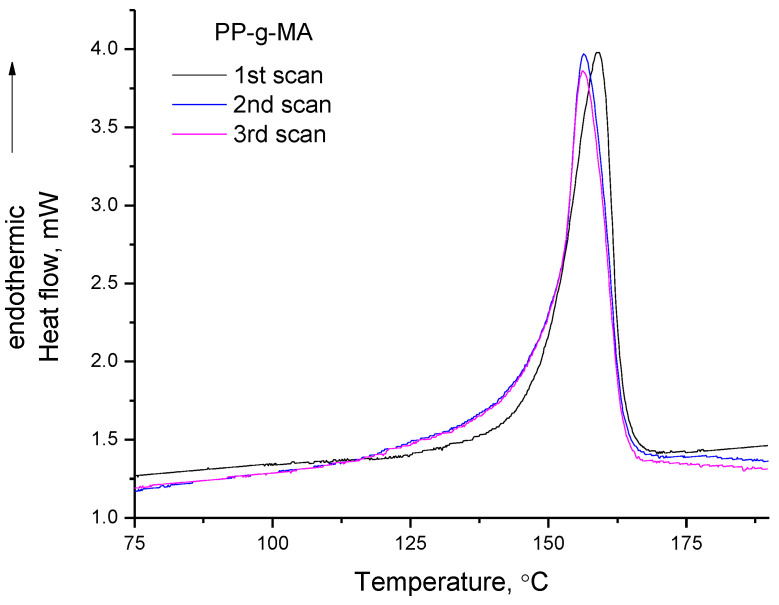
Three consecutive DSC scans of PP-g-MA.

The first TGA heating of PP-g-MA which was used in this study was presented recently in the discussion of LLoD [14] and, precisely, up to 180 °C mass loss of 0.37 wt.% occurs. This mass loss occurs at a temperature range which overlaps with the DSC peak of PP-g-MA. In the second and third TGA heating, no mass loss is detected (curves not shown). In Figure 3, the DSC curves of PP-g-MA for the first, second and third heating cycle are presented. Since only in the first heating of PP-g-MA mass loss was detected in TGA, this mass loss could be attributed to the evaporation/desorption of water and other impurities. However, such processes are endothermic, and the required heat should be hidden/overlapped by the “melting” peak. In addition, if the mass loss is attributed to factors such as the evaporation of impurities, well, it surely is a very interesting coincidence that the impurities vaporize at the same temperature range at which the polymer melts. However, as will be presented in the following sections, similar observations can be made for other polymers. Thus, it is difficult to accept that the impurities of PP-g-MA or PVA or PVC have boiling points close to the melting points of the respective polymers. Additionally, and more importantly, alterations in the chemical structure of PP-g-MA can be detected by FTIR. Before discussing the FTIR results for PP-g-MA, it is stressed that as in the case of PVA, and in all three DSC scans of PP-g-MA (Figure 3), the DSC peak is highly asymmetrical and also, the apparent heat capacity of the sample is decreased at the end of the “melting” peak compared to its values before the peak (simply this decrease is more mild compared to that of PVA, and is related to the lower extent of mass loss).

**Figure 4:**
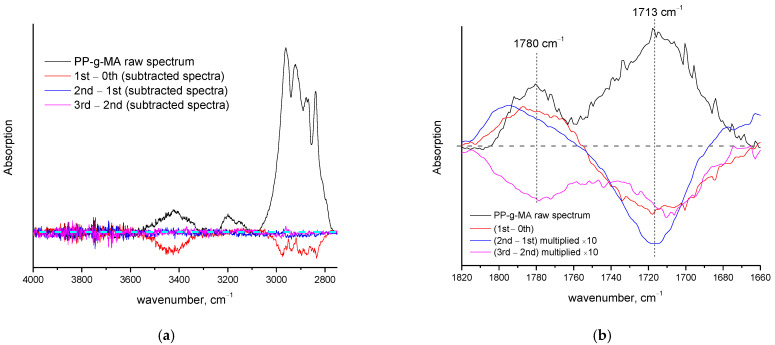
FTIR spectrum of PP-g-MA raw material and subtracted spectra before and after three consecutive heating cycles at 170 °C: (**a**) region 4000–2750 cm^−1^; (**b**) region 1820–1660 cm^−1^.

The FTIR spectrum of the raw (unheated) PP-g-MA sample and the subtracted spectra before and after the three consecutive heating cycles are presented in Figure 4 for two different wavenumber regions. As can be seen in Figure 4a, a negative peak of the CH_2_-CH_3_ bands at around 2900 cm^−1^ is clearly revealed in the 1st–0th subtracted spectrum, suggesting a decrease in organic substance after the first heating (a negative peak can also be detected in the 2nd–1st subtracted spectrum). Of course, the polar groups of maleic anhydride are expected to cause water absorption by the PP-g-MA, and thus the mass loss detected by TGA around 100 °C could be attributed mainly to water loss. However, in the DSC curve of the first scan, there is no detectable thermal effect around 100 °C. Thus, any removal of physically bounded water seems to occur at a very low extent and cannot be the primary cause for the mass loss detected in TGA. However, removal of chemically bounded water occurs. During production/storage of PP-g-MA, water absorption leads to the hydrolysis of maleic anhydride groups, which in turn results in the formation of maleic acid. Thus, PP-g-MA is actually polypropylene co-grafted with maleic anhydride and maleic acid, and not just maleic anhydride. This can be clearly detected by the FTIR measurements in the hydroxyl stretching region at 4000–3200 cm^−1^ (negative OH peak at around 3400 cm^−1^ in Figure 4a), but also in the carbonyl stretching region at 1700–1800 cm^−1^ (Figure 4b). The peak at 1713 cm^−1^ (characteristic of C=O of acids) becomes negative after the first heating, which indicates that acid groups have decreased. A positive peak at 1780 cm^−1^ (characteristic of C=O of anhydrides) has appeared, which indicates that new anhydride C=O groups have been formed (obviously related to the decrease in acid groups due to dehydration during the first heating cycle). The same trend, but at lower extent (to enable of visualization, spectrum multiplication was performed), is detected between the first and second heating (Figure 4b). Between the second and third heating a different behavior is revealed and along with the negative peak at 1713 cm^−1^, a negative peak at 1780 cm^−1^ has appeared, clearly suggesting a decrease in the anhydrides groups and the occurrence of decomposition through a different pathway than the one in the first heating.

**Figure 5:**
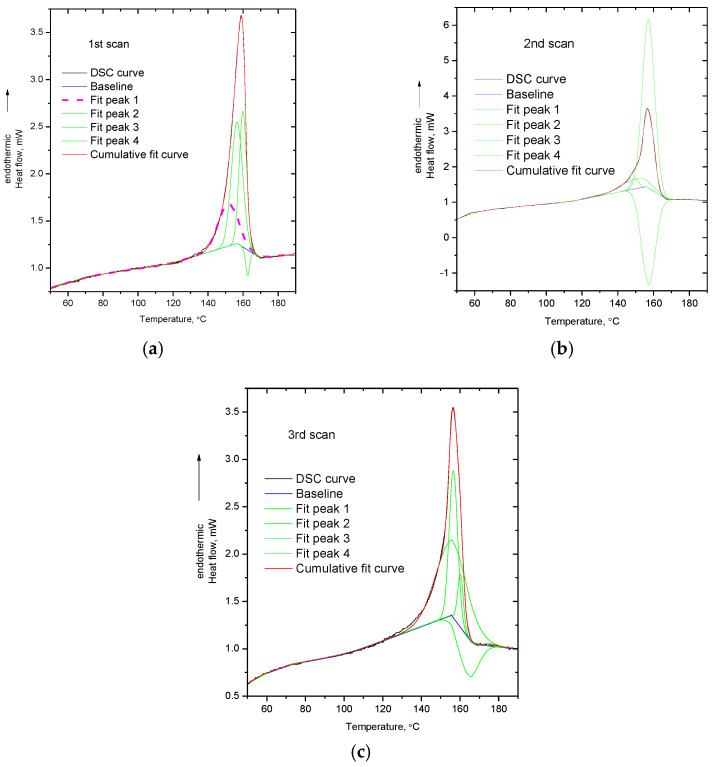
Fitting of the DSC curves of PP-g-MA: (**a**) first scan; (**b**) second scan; (**c**) third scan.

Although maleic acid should not be present in the structure of PP-g-MA, it is interesting that the chemical reaction of dehydration occurs upon “melting”. The anhydride groups can act as proton acceptors in a hydrogen bond, while the carboxyl groups can act both as donors and acceptors. Thus, maleic anhydride groups cannot form hydrogen bonds with each other, while acid groups can form hydrogen bonds with both anhydrides and other acid groups. In other words, the occurrence of dehydration results in a reduction in the number of possible hydrogen bonds, and this could enable “melting”. In addition, dehydration results in the production of small highly mobile molecules (water) which can act as plasticizers, i.e., increase polymer’s free volume and increase chain mobility (through collisions). Again, this facilitates the softening of the polymer. Consequently, since dehydration occurs in all three heating cycles and occurs simultaneously with “melting”, it is clear that the softening of PP-g-MA is a chemically induced phenomenon and overlaps with mass loss—that is, it is a thermochemical transition and not a melting point. To further explore the thermal behavior of PP-g-MA, the enthalpy of the dehydration reaction of maleic acid into maleic anhydride and water was calculated from the respective enthalpies of formation, and curve fitting of the DSC curves was also performed. The enthalpies of formation for (solid) maleic acid, (solid) maleic anhydride and (gas) water are equal to −789.4, −469.8 and −241.8 kJ/mol [37], respectively. From these values, the enthalpy of the reaction (dehydration of maleic acid into maleic anhydride and water) is estimated to be 77.8 kJ/mol. Although this value is higher than that of the esterification/hydrolysis reaction (16.4 kJ/mol) between glucose and acetic acid (resembling the reaction taking place upon heating of cellulose acetate) [10], it is rather low for a chemical reaction—the energy of some hydrogen bonds may be as high as 80 kJ/mol and thus this chemical reaction would be expected to occur easily. Additionally, this value (77.8 kJ/mol), if expressed per g of produced water, is translated to 4319 J/g. In Figure 5, the curve fitting (with four Gauss peaks) of the DSC peaks of the three scans of PP-g-MA is presented. As for the case of PVA, again, negative peaks are involved in the fitting in order to describe the asymmetry of the peak and the decrease in the baseline at the end of the peak.

The area of the first fit peak (shown with pink dashed line in Figure 5a) at 151 °C of the DSC peak of the first scan represents the 26% of the area of the cumulative peak. The cumulative peak corresponds to the value of 167.4 mJ as measured by DSC, thus the first fit peak corresponds to $\frac{26}{100}\times 167.4=44.2\;\mathrm{mJ}$. In the DSC experiment, the mass sample was 2.3 mg. From the TGA the % mass loss is known (0.37 ± 0.1 wt.%) [14]. By assuming that all this mass loss arises from water produced by the dehydration of acid groups, it follows that the mass loss during the first DSC scan was $2.3-\frac{0.37\times 2.3}{100}=0.00851\;\mathrm{mg}$. By dividing the value of 44.2 mJ with this mass loss and by taking into account the uncertainty of the % mass loss, a value in the range 4090–7125 J/g is obtained, which is of the same order of magnitude as the above mentioned theoretically calculated value of 4319 J/g. Similar calculations were not performed for the second and third scan, since no mass loss for these cases was directly detected by TGA.

Besides this endothermic peak, there are two more endothermic contributions and one exothermic in the DSC curve of the first scan (Figure 5a). However, no exothermic contribution is expected from the dehydration of acid due to the volatility of water. More specifically, at temperatures close to or within the softening point of PP-g-MA, i.e., temperatures much higher than 100 °C, any produced water is expected to rapidly vaporize and thus the reverse (exothermic reaction) of hydrolysis of anhydride groups by water is not expected to proceed at any countable rate. In addition, if dehydration was the main cause for the asymmetry of the DSC peak of PP-g-MA, then this should mainly be observed in the first scan, since in the other two heating cycles the extent of dehydration is 10–20 times lower (justification for this is provided below). In combination with the 3rd–2nd FTIR subtracted spectrum (negative peak at 1780 cm^−1^ indicating decrease in anhydride pendant groups) and the 1st–0th FTIR subtracted spectrum (negative in 2900 cm^−1^ indicating reduction of backbone C-H groups) it seems that additional decomposition reactions take place along with dehydration. Thus, as for the case of PVA, the exothermic contributions in all three DSC curves, as well as the asymmetry of the DSC peaks, can be related to decomposition.

Finally, it is worth mentioning the following:

(1) From the FTIR (Figure 4b) it can be seen that the area of the negative peak at 1713 cm^−1^ (acidic C=O stretching) is almost the same in the 1st–0th subtracted spectrum and the 2nd–1st subtracted spectrum. In other words, the decrease in acidic groups is the same, however, the 2nd–1st subtracted spectrum was multiplied by 10. Thus, the reduction of acidic groups (or the extent of dehydration) in the second heating is about 10 times smaller than that in the first heating. Accurate calculation of the areas of the negative peaks yielded a ratio of 8.8 (in other words, the decrease in acidic groups is 8.8 times smaller in the second scan). Thus, in the second scan the % mass loss due to dehydration is estimated to be $\frac{0.37\%}{8.8}=0.042\;\mathrm{wt}.\%$. This value is very close to the LLoD value for PP and PP-g-MA (measured under nitrogen atmosphere) which has been reported to be ~0.03 wt.% [14]. The respective ratio of the areas of the negative peak at 1713 cm^−1^ in the 2nd–1st subtracted spectrum and 3rd–2nd subtracted spectrum is 2.2 (the mass loss due to dehydration in the third scan is 2.2 times smaller than the one of the second scan) corresponding to 0.019 wt.% mass loss due to dehydration in the third heating. Thus, based on LLoD of TGA [14], it can be perfectly justified why no mass loss is detected in the second and third TGA heating, despite the fact that dehydration occurs in all three heating cycles as confirmed by FTIR.
(2) In the second and third heating cycles, it is more difficult for dehydration to occur due to the smaller number of hydrogen bonds. A lower number of acidic groups is translated to a decreased number of bounded acid groups and an increased number of free ones. The free acid groups are stronger compared to the hydrogen-bonded groups since hydrogen bonding weakens the O-H chemical bond. The chemical reaction of dehydration of acid requires the breakage of O-H chemical bond. Thus, in bounded acid groups the reaction is facilitated, since it is easier to break the O-H chemical bond due to its weakening. In the third heating, the number of acidic groups is lower and the % portion of free groups is expected to increase. Consequently, dehydration occurs at even lower extent.
(3) Maleic acid, which is present in the PP-g-MA as a result of the hydrolysis of maleic anhydride groups, is chemically similar to succinic acid which is suspected to exhibit a “problematic” thermal behavior [5].

### 3.3. Isotactic PP

**Figure 6:**
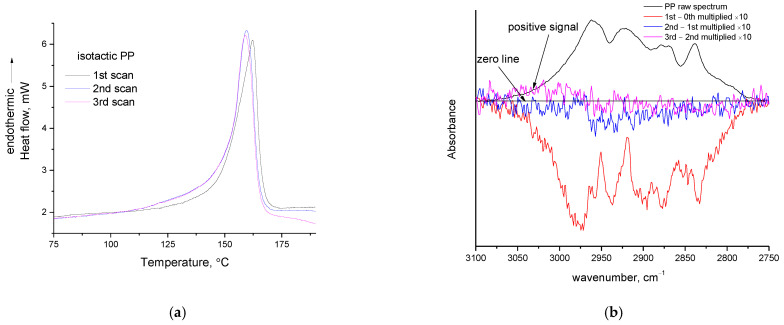
(**a**) Three consecutive DSC scans of isotactic PP sample; (**b**) FTIR spectrum of PP raw material and subtracted spectra before and after three consecutive heating cycles at 170 °C in the region 3100–2750 cm^−1^.

The three consecutive DSC scans and the FTIR spectra of PP are presented in Figure 6a and Figure 6b, respectively. In none of the three consecutive TGA heating cycles of PP was mass loss detected (curves not shown). Regarding the DSC curves, the same observations can be made for PP as for PVA and PP-g-MA; that is, an overall asymmetry in the peak and a decreased heat capacity after the “melting” peak. Again, this can be interpreted as a contribution of energy release during the formation of new bonds from non-volatile decomposition products. Curve fitting for these DSC curves is presented in the supporting information (see Figure S4). In the DSC traces of PP besides the “melting” peak, no other thermal effect can be detected. Thus, the heat involved in this endothermic peak must be responsible for the negative peaks realized by FTIR (Figure 6b). In addition, since there is no other thermal effect detected in DSC, that could justify the negative peaks; these negative peaks after the first heating cannot be attributed to the evaporation of impurities (unless if it is accepted that the previously mentioned coincidence holds, that is, that the impurities of PP have similar boiling point with the melting point of PP).

**Figure 7:**
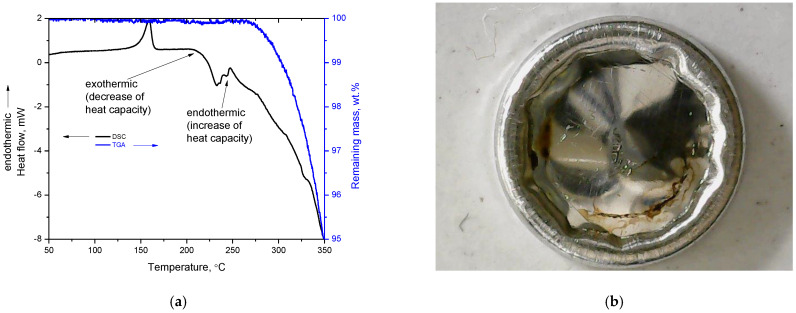
(**a**) DSC and TGA curve of isotactic PP up to 350 °C; (**b**) Photograph of the DSC pan at the end of the measurement up to 350 °C revealing the actual mass loss (brown/black color) which has occurred during the measurement.

A negative peak in the C-H region (2750–3000 cm^−1^) can be detected after the first heating and after the second heating (but with a very low, close-to-noise intensity). After the third heating, the appearance of a very small positive peak in the region 3100–3200 cm^−1^ (indicated by arrow in Figure 6b) seems to occur. As for the case of PVA [12], this may be related to the formation of carbon–carbon double bonds (“char” regions). Of course, in the case of PP, the intensity is very low even after spectrum multiplication, thus the C=C content should be extremely low. In a later section, it will be theoretically justified why PP is expected to exhibit a very low extent of decomposition during its softening. Based on the above, it seems likely that isotactic PP exhibits a thermochemical transition, but the extent of decomposition during softening is hardly detectable. Additionally, in one study in the literature, the decomposition of isotactic PP was studied, and the first decomposition products were detected by Mass Spectrometry at 177 °C—a temperature very close to its melting point (7 °C above the melting point)—the authors speculated that such a low temperature was “probably slightly in error” [38]. In the same study, by FTIR spectroscopy, the first decomposition products were detected at 217 °C [38]. Besides these, further insights can be provided by the DSC and TGA curve of PP up to 350 °C (Figure 7a).

As can be seen in Figure 7a, around 250 °C, an endothermic peak appears which overlaps with the initiation of mass loss in TGA. This endothermic contribution can be related to the sensible decomposition detected in TGA. The DSC baseline after this temperature range is highly exothermic and is governed by the mass loss whose occurrence is clearly detected in TGA. The actual mass loss out of the DSC pan is confirmed by the photograph of the DSC pan after the measurement up to 350 °C, which is presented in Figure 7b. The slope of the DSC baseline is less negative compared to the mass loss slope in TGA (Figure 7a), since heat is continuously absorbed by the sample and this tends to shift the DSC baseline towards the endothermic direction. However, as mentioned above, PP is known to decompose at lower temperatures, i.e., in the range 177–217 °C [38], and in the DSC curve (Figure 7a) an intense exothermic shift of the baseline is clearly visible at around 220 °C. As discussed numerous times throughout the text, the exothermic shift is related to decomposition (energy release from reactions among the decomposition products). In addition, DSC has no similarity to TGA limitation (LLoD) in detecting mass loss due to a sample’s buoyancy, since, in contrast to TGA, the DSC sensor is influenced by the real mass of the sample inside the pan and not from the apparent weight of the sample. Thus, if actual mass loss out of the DSC pan occurs, this would be directly detected by DSC, since less heat would be required in order to keep the same rate of temperature increase in the sample and in the reference (empty) pan. This mass loss would not be detected by TGA until it became high enough to surpass the sample’s buoyancy [14]. However, besides the “melting” peak, there is no other effect which can be responsible for the intense exothermic shift at 220 °C.

Based on all the above, it is concluded that in the “melting” peak of PP there is a considerable contribution for heat required for decomposition; that is, the softening of PP is a thermochemical transition.

### 3.4. PVC

**Figure 8:**
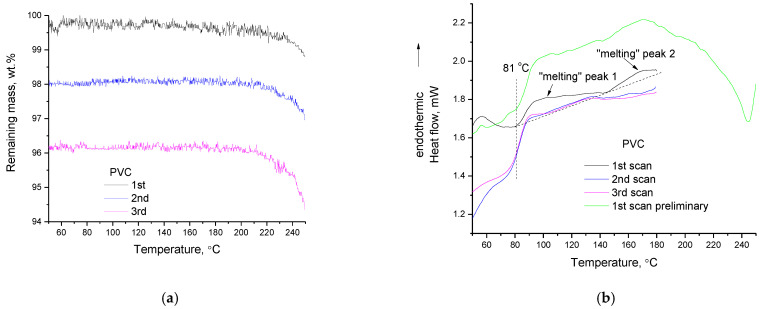
(**a**) Three consecutive TGA heating cycles of PVC up to 250 °C. The green line points out a decreasing trend of mass in the range 125–200 °C in the first heating; (**b**) Three consecutive DSC scans of PVC up to 180 °C and a preliminary first scan up to 250 °C. The dashed line has been drawn to enable the recognition of the peaks.

In Figure 8a, the three consecutive TGA heating cycles for PVC are presented. It can be seen that PVC decomposes at temperatures above 200 °C. In Figure 8b the three consecutive DSC scans of the PVC sample (along with one preliminary DSC scan) are presented. An intense increase in the heat capacity initiates at 81 °C (in the first scan) and another one initiates to occur around 140 °C. In the literature [39,40], these curves are considered as melting peaks (indicated with arrows in Figure 8b—also in the same Figure, a dashed baseline has been drawn to enable recognition of the peaks) and from these peaks, the heats of fusion and the degree of crystallinity are estimated. In a very interesting review article regarding the crystallinity of PVC [41], various DSC and X-ray diffraction (XRD) graphs of PVC are presented. These DSC curves are very peculiar, like the ones presented in this study. PVC in general has a diffuse broad XRD pattern and as it has been reviewed [41], it exhibits a low degree of crystallinity of the order of 10%; however, this crystallinity is of a different nature from that of other polymers and thus it has a significant impact on the PVC’s properties. The two “melting” peaks have been attributed to two different crystallite types, namely fringed micelle type and lamellar type [40]. Furthermore, although polymers in general do not give sharp but rather broad asymmetric melting peaks in DSC, the broadness and asymmetry of the “melting” peaks of PVC are very extensive, e.g., the first peak covers a range of 80–140 °C (Figure 8b).

This inconsistency (low XRD signal but rather intense DSC signal) is similar to the inconsistency [42] regarding the conclusion about the crystallinity of cellulose esters as derived by XRD and DSC that was recently clarified [9] along with rather unusual interpretations, e.g., that once melted they cannot be recrystallized by thermal treatment, but only after solvent precipitation. On one hand, it seems reasonable that two different types of crystallites would be responsible for two melting peaks, but on the other hand, how is it possible for PVC, a polymer with such a low crystallinity (as implied by XRD), to exhibit not one, but two melting peaks in the DSC? The behavior is exactly the same as that of CAB and CA (that is, a transition appearing as glass transition with a small endothermic peak at its end). In the third scan of PVC, the small peak at the end of the “glass transition” is clearly visible and more easily realizable. In general, this peak is commonly attributed to enthalpy recovery upon heating, caused by the enthalpy relaxation that occurs in the polymer when it is stored at lower temperatures but rather close to the glass transition temperature, so an adequate chain mobility will exist in order to result in relaxation/rearrangement. However, in the DSC scans which were performed in this study, the cooling rate was low (room temperature cooling), and no quenching was applied. Thus, there should not be any considerable tendency for enthalpy relaxation and thus enthalpy recovery. In addition, between the consecutive scans there is not enough time for enthalpy relaxation to occur. Thus, this peak at the end of “glass transition” should not and cannot be interpreted as enthalpy recovery peak.

If it is assumed that PVC does not exhibit a thermochemical transition, then, as for PP, the following inconsistency between DSC and TGA arises: From the TGA curves (Figure 8a) it is apparent that considerable mass loss occurs in PVC at temperatures above 200 °C. Obviously, heat is needed to break the chemical bonds and decompose PVC. However, in the DSC curves above 200 °C (preliminary first scan in Figure 8b), no endothermic effect is detected and, on the contrary, the DSC signal is shifted towards the exothermic direction (this exothermic shift initiates at around 170 °C). If the peaks of DSC are considered to be melting peaks, then where is the heat required for decomposition? Why does DSC not detect the rather high amount of heat required to induce decomposition of 1–2 %wt. as detected in TGA? This inconsistency can be easily solved by accepting that PVC does not exhibit melting points but thermochemical transition. More specifically, the heat required for decomposition is detected by DSC at temperatures lower than 200 °C. The exothermic shift of the DSC signal (that initiates at 170 °C) is related to a decrease in heat capacity due to heat release caused by the formation of new chemical bonds from the reactions among the early-stage non-volatile decomposition products. Additionally, note, that the PVC used in this study is of a very high molecular weight (as stated by the manufacturer). Thus, it is more likely at early stages to produce large, non-volatile fragments. TGA does not detect mass loss at this temperature range due to lack of volatility of the decomposition products. In addition, even after the formation of volatile decomposition products, TGA will not detect any mass loss, unless the LLoD is surpassed [14]. As the temperature increases, e.g., at 200 °C, two factors contribute to the mass loss: a) decomposition proceeds (both endothermic and exothermic reactions) but the portion of volatile decomposition products is increasing since smaller (more volatile) fragments are likely to be produced and b) the vapor pressure increases, meaning that some products that could not vaporize at 170 °C can vaporize above 200 °C. Thus, above 200 °C, mass loss increases and is detected by TGA. This mass loss is also detected by DSC as further shifting (at around 210 °C) of the DSC signal towards the exothermic direction because of a decrease in heat capacity due to a decrease in mass sample.

**Figure 9:**
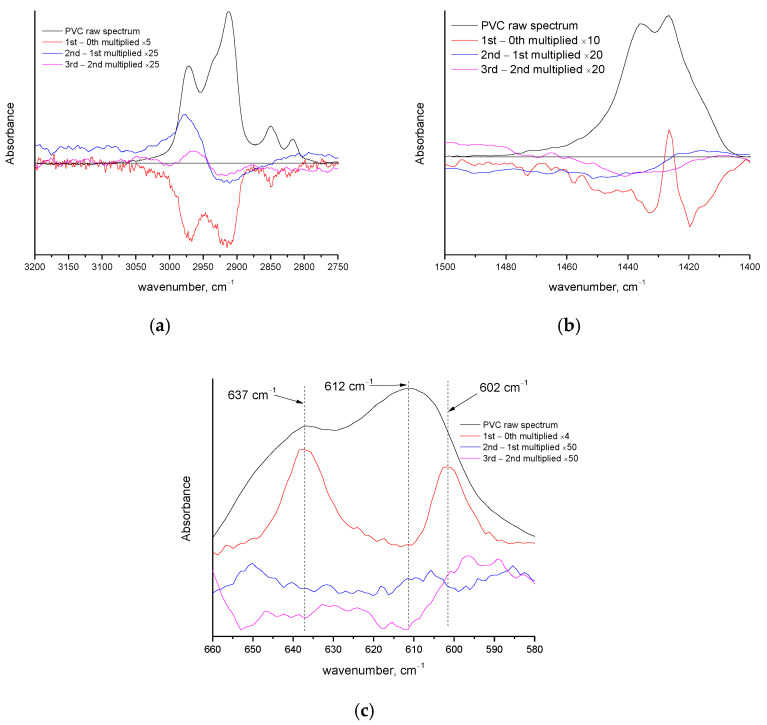
FTIR spectra of PVC raw material and subtracted spectra before and after three consecutive heating cycles at 170 °C: (**a**) in the region 3200–2750 cm^−1^; (**b**) in the region 1500–1400 cm^−1^; (**c**) in the region 660–580 cm^−1^.

The occurrence of decomposition of PVC at temperatures up to 170 °C is also confirmed by the FTIR measurements (Figure 9). The absorption around 2900 cm^−1^ (C-H stretching vibrations) is negative after the first heating (Figure 9a). In other regions of the spectrum some positive peaks have appeared. More specifically (Figure 9b), the absorption detected in this work at 1426.5 cm^−1^ (literature value [43] at 1424 cm^−1^) is attributed to CH_2_ symmetric (scissors) deformation in the crystalline phase and has become positive, while the absorption of the respective vibration of the amorphous phase at 1435 cm^−1^ (literature value [43] at 1431 cm^−1^) is zero or slightly negative. These suggest a slight increase in crystallinity. A similar conclusion is derived from the C-Cl vibrations (Figure 9c). The C-Cl stretch in the amorphous phase at 612 cm^−1^ (literature value [43] 610 cm^−1^) is slightly negative after the first heating, while a new positive peak has appeared at 602 cm^−1^ (literature value [43] at 603 cm^−1^), which is the corresponding vibration in the crystalline phase. The overall absorbance of the multiple peaks related to C-Cl at around 620 cm^−1^ (A_620_) is increased with respect to the absorbance at 2900 cm^−1^. More specifically, the ratio A_620_/A_2950_ increased from 2.9 in the raw sample to 3 after the first heating; however, a similar increase is observed for the ratio A_1430_/A_2950_ that increased from 0.7 to 0.8. The comparison of C-Cl and CH_2_ in the regions at 620 cm^−1^ and 1430 cm^−1^, respectively, that are both influenced by the relative changes in crystallinity, can be considered more reliable. The ratio A_600_/A_1430_ decreased from 4.1 in the raw material, to 3.8 after the first heating, suggesting a decrease in the overall (amorphous and crystalline) C-Cl with respect to the corresponding (amorphous and crystalline) CH_2_ groups and these suggest a pendant group decomposition pathway. The overlapping of the vibrations of C-Cl and CH_2_ in the crystalline and amorphous phases does not allow us to distinguish a similar decrease in amorphous contribution and increase in crystalline contribution between the first and second and the second and third heating cycles. However, in the region 2750–3000 cm^−1^ (Figure 9a) both positive and negative peaks can be detected even after third heating, suggesting alterations in chemical structure. Finally, it is worth mentioning that the pendant group elimination mechanism for PVC decomposition is in agreement with the literature [44,45] and is related to HCl production. Although this is a volatile substance, its low molecular weight may also contribute to the absence of severe mass loss at the early stage of PVC decomposition.

### 3.5. PS

**Figure 10:**
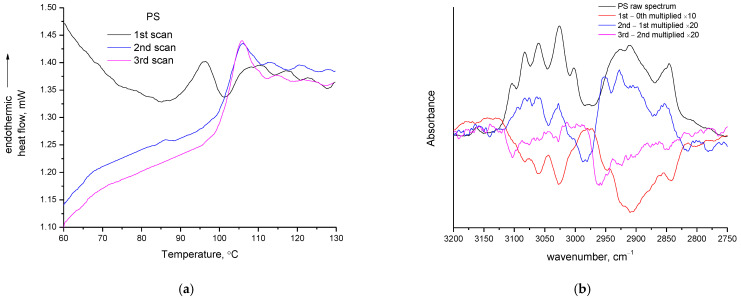
(**a**) Three consecutive DSC scans of the PS sample; (**b**) FTIR spectrum of PS raw material and subtracted spectra before and after three consecutive heating cycles at 130 °C in the region 3200–2750 cm^−1^.

As for the case of PP, and also in the case of PS, no mass loss could be detected in the three TGA heating cycles (curves not shown). However, in the DSC traces of PS (Figure 10a) decomposition can be realized. In all three scans an intense increase in heat capacity initiates around 85–90 °C and ends around 105 °C. The above mentioned (for PVC) small endothermic peak at the end of “glass transition” is clearly visible in all three scans of PS. The major difference between the three scans is that in the first scan, the increase in heat capacity from 85 to 105 °C is not continuous and a severe decrease occurs in the range 95–100 °C, and afterwards an intense increase occurs again. The decrease in heat capacity makes the signal look like an endothermic peak. The decrease in the first scan is difficult to explain in terms other than decomposition. It is either an endothermic decomposition peak or it is a decrease in the heat capacity due to exothermic reactions of non-volatile decomposition products (or most likely a combination of such overlapping effects). For similar reasons to those already discussed for PVC, the interpretation that the peak at the end of “glass transition” is related to enthalpy relaxation is highly disputable. This behavior is the same as that of CAB [9].

From the FTIR (Figure 10b), a reduction in both CH_2_ stretch (around 2900 cm^−1^) and aromatic CH stretch (around 3050 cm^−1^) is realized after the first heating at 130 °C. After the second heating, both absorptions become slightly positive and this could be attributed to density increase due to physical factors (e.g., PS is completely amorphous and consequently there is no equilibrium state, and thus, by heating and cooling, the chains may arrange in slightly denser manner). However, the CH_2_ absorption (which is less intense in the raw spectra compared to the aromatic CH absorption) becomes slightly more positive than that of aromatic CH. The relative difference in these two absorptions suggest alterations of density due to chemical (and not just physical) factors. Between the second and third heating, slight decreases in both absorptions seem to occur. Finally, the impurities cannot be the cause of the decrease in the heat capacity and the alterations of the FTIR spectra, unless it is accepted that the impurities of PS have boiling points similar to the glass transition temperature of PS, and for some reason they are not removed after the first heating.

### 3.6. Discussion on the Effect of Impurities

As mentioned several times throughout the text, the observed behavior cannot be attributed to impurities. If this was observed for one polymer, then the matching of the boiling point of the impurity with the melting point (or glass transition) of the polymer could be considered a coincidence. However, this is observed in too many polymers (the ones presented in this study, and also in CAB and CA). Even if the mass loss is attributed to impurities, it is obvious that the removal of impurities and the softening of the polymer are not independent, otherwise they should occur at different temperatures. Water is an impurity for PP-g-MA, yet it seems to play an important role in its thermal behavior. However, as discussed, PP-g-MA also decomposes through another pathway. Thus, the presence of the impurity may intensify the “problem” but is not the primary cause. For gallic acid, it was reported that the presence of different solvent impurities affects the decomposition pathway, and higher or lower mass loss can occur during the solid–solid thermochemical transition around 90 °C, but the presence of impurities is not the primary cause for the thermochemical transition [17]. Similarly, composite membranes of CA with gallic acid or quercetin exhibited depressed thermochemical transition temperature but higher extent of decomposition during softening compared to pure CA membrane [11]. If gallic acid and quercetin are considered as impurities for CA, then it is apparent that their presence intensifies but does not cause the thermochemical transition of CA. These polymers are unable to actually melt (i.e., soften without decomposing). For polymers in general, there are various potential decomposition pathways/reactions and obviously the easiest pathway (thermodynamically and kinetically favored) will prevail. The impurities may favor a specific decomposition pathway, e.g., the dehydration of acid groups of PP-g-MA due to low enthalpy of reaction but are not the primary cause for the thermochemical transition. Additionally, the matching of the boiling point of an impurity with the softening point of a polymer, which will be discussed in the next section, may be apparent and may arise from factors other than the actual presence of impurities.

## 4. Theoretical Considerations and Discussion

### 4.1. Mobility of Pendant Groups and Some Interesting Coincidences

In polymers, the mobility of the high length backbone is lower than that of the small pendant groups at any given temperature (except extremely low temperatures where the mobility of pendant groups is also low). A similar statement can be made for the end groups that are chemically bonded to the backbone only from one side. This large difference in the mobility between the groups/bonds within the same molecule can be considered as a general characteristic of high molecular weight substances and thus, it should play a determining role in the properties of polymers. The influence of the increased mobility of the pendant groups can be recognized, in some cases, from the obvious matching of the boiling point of the pendant groups and the thermal transition temperature of the polymer. The primary trigger for the work on CAB [9] was the observation that the believed “melting” point of CAB was suspiciously close to the boiling point of butyric acid. Additionally, the “glass transition” temperature of CAB (depending on the overall and partial degrees of substitution) is suspiciously close to the boiling point of the pendant groups/possible decomposition products such as butyric acid and acetic anhydride. In cellulose esters besides thermal decomposition, chemical decomposition through hydrolysis also occurs [9] leading to acid formation and complicating the interpretations; however, the above mentioned matching can be realized in other polymers where there is no involvement of hydrolysis. For PS, the “glass transition” in the first scan starts to occur at 85 °C, which is just above the boiling point of its pendant groups (benzene), which is 80 °C. In the second and third scan the onset temperature is higher (~95 °C) but it can still be considered to be close to the boiling point of benzene. Benzene, as a pendant group, is of large volume; thus, it governs the distance among chains and makes the contribution of the backbone hydrogen atoms’ interactions negligible. In PS, the benzene groups are found in every repeating unit and interact with each other. Although chemically bonded to the backbone at one point, the other five of the six C atoms (and the corresponding H atoms) are found in an environment not too different from that found in liquid benzene (that is, the benzene groups of the polymer interact with each other with similar intermolecular forces as in the liquid state of benzene). Thus, for a polymer like PS (low polarity backbone, pendant groups of high volume and increased number of pendant groups), at the temperature at which the energy/mobility of (free) pendant groups is increased to the point that it would normally boil, it is perfectly reasonable for a similar (distorted) phenomenon to occur with a tremendous impact on the polymer’s behavior. Decomposition/distorted boiling enables any thermal transition/relaxation of the polymer, by increasing (to different extents) the free volume of the polymer and by enhancing the mobility of the backbone (through the extensive thermal motion of pendant groups). Additionally, the (volatile) decomposition products may act as plasticizers that further enable relaxation.

As stated above, the end groups, like the pendant groups, also have increased mobility compared to the backbone groups. The same seems to hold for other small distinguishable parts of the backbone, such as the actual repeating unit or oligomers (2–5 repeating units), since a matching of the boiling point of such distinguishable fragments of the backbone and the polymer’s thermal transition temperature can be recognized. It is stressed that the term “actual repeating unit” is used instead of “monomer”, since these two do not always coincide. A typical example is the polyester named poly(L-lactic acid). The pendant groups of PLLA are methylene and carbonyl oxygen. Methyl acetate (boiling point 56.8 °C) can be considered as the simplest organic substance to resemble the “actual” repeating unit and the pendant/end groups of PLLA and, just above its boiling point, PLLA exhibits a glass transition (~62 °C).

In the introduction section, the broadness and the asymmetry of the melting peaks of polymers was discussed. In the DSC curves presented in the previous section, this asymmetry of the “melting” peaks can be detected as a tail/shoulder in the beginning of the peaks. For the case of PP, the tail seems to initiate in the range 110–125 °C. 2,4-dimethyl-hexane (a distinguishable part of the backbone) has a boiling point of 109 °C. Other (linear and branched) alkanes (C_9_-C_10_) have boiling points in the temperature range 120–175 °C. As mentioned in a previous section (Section 3.3), in one study in the literature, the first decomposition products of isotactic PP were detected by Mass Spectrometer at a temperature just 7 °C above its melting point [38]. In the decomposition products, higher molecular weight species (C_5_-C_7_) were detected [38]. In any case, the random scission decomposition pathway of PP and the boiling point of PP’s dimer/trimer are in agreement with the tail of the peak and the decomposition after 125 °C that is interpreted from DSC. The pendant (methylene) groups of PP are small, do not exhibit strong interactions and although there is an increased number of pendant groups (one pendant group per repeating unit), in contrast to benzene and the case of PS, the environment is quite different to that in the liquid state of methane (the simplest compound to resemble the pendant group of PP). Thus, in such a case, a direct matching of the boiling point of the pendant group and the thermal transition of the polymer would not be expected.

The partial overlapping of PP-g-MA’s “melting” region with the melting point of maleic acid (129–145 °C) does not seem likely to be accidental; however, as discussed, a decomposition pathway seems to exist in addition to dehydration. For this second decomposition pathway, a similar discussion to that for the case of PP also holds for the case of PP-g-MA.

For the case of PVA [12], it was recently pointed out that its “glass transition” is also a thermochemical transition and occurs very close to the boiling temperatures of methanol and ethanol. The simplest compounds to resemble the pendant or end groups of PVA are alcohols like methanol (boiling point ~65 °C) and ethanol (boiling point ~78 °C).

For PVC, the labile nature of the C-Cl bond governs its thermal transition, but for the reasons explained, like with PP, no direct matching would be expected. Additionally, it is noted that similar matchings have been recently reported for various vinyl esters [10].

Based on the results presented up to this point and the overall discussions, it is proposed that the simultaneous softening and decomposition in polymers arises from a distorted version of an actual first-order transition, i.e., boiling. In cases where a large number of pendant groups exist, and depending on other factors such as size, strength of physical interactions, etc., the thermal transition of the polymer may not be at all a distorted, but instead a clear version of the boiling of the pendant groups. In other cases, such as cases with two different kinds of pendant groups or a small number of pendant groups— although the boiling point of the pendant groups is not readily recognized—above some temperature (or temperature range), their mobility increases considerably, and this distorted boiling causes the thermochemical transition (simultaneous softening and decomposition) of the polymer.

### 4.2. Thermal Transition Behavior of Polymers under a Unified Basis and Latent Decomposition

Up to this point, it was discussed that: (1) various common polymers exhibit thermal transitions that occur simultaneously with decomposition and (2) the increased mobility (distorted boiling) of the pendant/end groups/small backbone segments seems to be related to this effect. It is more reasonable and physically meaningful for thermoplastic polymers to exhibit thermal behavior, more common of other polymers, such as thermosets, rather than other low molecular weight substances. It is proposed that the simultaneous softening and decomposition is a general property of polymers. More specifically, it is proposed that polymers cannot be softened without being decomposed, and the only difference, among thermosetting polymers, biopolymers and thermoplastics, is the extent of decomposition or, in other words the relative contributions of softening and decomposition during thermochemical transition. What follows is an attempt to theoretically support this idea. In essence, basic concepts of the kinetic gas theory (e.g., random collisions) are applied to the pendant and end groups of the polymers.

Upon heating, not all of the pendant (or end) groups have the same mobility/energy, due to the randomness of collisions and therefore they randomly exchange energy with each other. An analogy can be drawn here between this and the boiling of a liquid. A small number of bubbles are formed much before the whole liquid mass boils. Such high energy bubbles (molecules with enough energy to form a stable vapor phase) will escape from the liquid upon heating. In the case of polymers, a small quantity of pendant groups (resembled by the initial bubbles) will have more energy than others at any given time. However, the high energy pendant groups are chemically bonded to the backbone and cannot escape as easily as the initial bubbles can from the liquid. By increasing temperature, due to the more frequent collisions and exchange of energy, the mobility (energy) of some of the pendant (or end) groups becomes so high that they can cause decomposition, either by detaching themselves from the backbone (mechanism of decomposition: either pendant group elimination or depolymerization) or by causing the breaking of the backbone bonds (mechanism of decomposition: random scission or depolymerization).

Cellulose is a typical example of a polymer that does not exhibit any detectable thermal transition prior to decomposition. The explanation for this behavior is the existence of an extensive inter- and intra-molecular hydrogen bond network. In general, the energy of a chemical bond is orders of magnitude higher than the energy of physical interactions. However, in the case of cellulose, upon heating, some chemical bonds are broken before a loosening of the physical interactions is accomplished. An attempt to explain this behavior in terms of statistical thermodynamics will be made.

According to Boltzmann’s thermal distribution, high energy states are less populated than low energy states and the probability of finding a molecule (pendant/end group in this case) with a certain energy equals the fraction of molecules with this energy. Here, two energy states will be considered for the mobility of the pendant groups of polymers during a thermal transition: (1) one low energy state corresponding to softening, and (2) one high energy state corresponding to decomposition. Before proceeding, the following should be defined:

N_D_: number of pendant groups per repeating unit which are found in the high-energy (decomposition) state during thermal transition/softening.

N_S_: number of pendant groups per repeating unit which are found in the low-energy (softened) state during thermal transition/softening.

(1) $$r=\frac{E_{CHEMICAL}}{E_{PHYSICAL}},$$

where:

*E_CHEMICAL_*: Is the energy of the weakest chemical bond of the polymer (bond other than the C-H bond).

*E_PHYSICAL_*: Is the energy of the strongest physical interaction among pendant groups of the polymer.

From the above it follows that the degree of apparent decomposition (*d*) equals

(2) $$d=\frac{N_{D}}{N_{D}+N_{S}},$$

The term “apparent” is used for decomposition, because the *N_D_*, is not the actual number of the pendant groups that will decompose, but the number of pendant groups with energy high enough to cause decomposition. In order to describe the proposed concept with a simple equation, it is assumed that the ratio of the probabilities for softening and decomposition state (and thus the ratio *N_S_/N_D_*) is proportional to the ratio of the energy of chemical bond to the energy of physical interactions, that is:

(3) $$\frac{N_{S}}{N_{D}}=a\times r,$$

where *a*: constant.

For simplicity, it is assumed that *a* = 1. From Equation (1) it follows that if there is a poor physical interaction then r will be high. From Equation (3) it then follows that, in such a case, the *N_S_/N_D_* ratio is also high which is translated to high *N_s_* and low *N_D_*; that is, high contribution of softening and low contribution of decomposition. In the opposite case, e.g., for cellulose, the physical interactions are too strong and there are too many, approaching the order of magnitude of chemical bonds, that is, the value of r is very low. Consequently, the *N_S_/N_D_* ratio is also low which is translated to low *N_s_* and high *N_D_*, that is, low contribution of softening and high contribution of decomposition. So based on these, the explanation for the behavior of cellulose, in terms of statistical mechanics, may be the following: The difference in energy for the two states is small, therefore the difference in the probability of finding a pendant group in each state is also small. This means that the two states are more uniformly populated, and the contributions of softening and decomposition tend to become comparable. For the degree of apparent decomposition *d*, from Equations (2) and (3) the following expression is extracted:

(4) $$d=\frac{1}{1+r},$$

This concept does not take into account the influence of the pendant atoms (C-H), the size of the pendant groups, tacticity, the effect of the end groups, the temperature variation of the strength of chemical and physical bonds, differences in intra- and inter-molecular interaction, weakening of chemical bonds due to strong physical molecular interactions, etc., but it is sufficient for the purposes of the current work to describe qualitatively and provide some elementary theoretical support for the idea of the unified thermal transition behavior of polymers.

In Table 1, the values of *E_CHEMICAL_* [37] and *E_PHYSICAL_* that were used for the calculations, along with calculated values of *r*, %*d* from Equations (1) and (4) and the % decomposition from the first TGA heating (for each polymer in the temperature range of the thermal transition) are presented. For the case of PP and PS where mass loss was not detected in TGA, the mass of the sample used in the TGA measurement and an uncertainty of 0.02 mg were used to calculate the minimum detectable decomposition. Besides the five polymers that were experimentally studied, three more (hypothetical) cases were examined: a hypothetic polymer to resemble cellulose and two hypothetical thermosetting polymers. Except for the case of PVC where the weakest chemical bond is C-Cl, for all other cases for the value of *E_CHEMICAL_* the energy of the C-C bond was used. For the *E_PHYSICAL_*, a value of 4 kJ/mol was used for the Van der Waals forces for PP, PS and PVC, and a value of 25 kJ/mol for the hydrogen bond in PVA. The C-H and C-Cl physical interactions (for the case of PVC) were given the same value, for simplicity, since the result for PVC is governed by the much lower *E_CHEMICAL_* value. For the case of PP-g-MA (with 1% MA content) the value of 0.99 × 4 + 0.01 × 20 = 4.16 kJ/mol was used. The value of 20 kJ/mol was used suggestively to resemble the high polarity of the maleic anhydride molecule. To resemble cellulose, *E_PHYSICAL_* was set to 75 kJ/mol (3 × 25) since there are three hydroxyl groups per repeating unit. Finally, for the thermosetting polymers, it was assumed that the pendant groups are chemically bonded to each other (crosslinked) and thus two much higher values for *E_PHYSICAL_* were used, in order to resemble the higher energy of the chemical crosslinking.

As can be seen in Table 1, the theoretical calculations indicate that polymers like PP and PS exhibit the lowest decomposition, while polymers like PVC (with more labile chemical bonds) or PP-g-MA (with increased polarity and thus increased physical interaction energy) are expected to exhibit higher decomposition. These are in agreement with the experimental TGA measurements for PP-g-MA and PVC (in the first heating the mass loss is high enough to be realized, in contrast to the cases of PP and PS). A comparison of the rest of the polymers with PVA also reveals an agreement between the theoretical calculations and experimental observations. The strong physical interactions due to hydrogen bonding in PVA are one order of magnitude higher than the interactions in PS, PVC, etc. The theoretical calculations indicate that PVA exhibits decomposition at least one order of magnitude higher than the one of other polymers, and this was also observed in the TGA measurements. By increasing the energy of the physical interactions to resemble cellulose, the theoretical calculations reveal an extent of decomposition during the softening point one order of magnitude higher than PVA, which is a severe decomposition, in agreement with reality. The extent of decomposition, by further increasing the physical interactions of pendant groups in the order of magnitude of chemical bonds, becomes, as expected, even higher. For the hypothetical case of a thermosetting polymer where the chemical bonds and the “physical” interactions are the same, a 50% decomposition is estimated, or in other words, the probability/contribution of softening and decomposition is the same.

If a distorted version of boiling of the pendant groups occurs in polymers at the thermal transition temperatures then, at lower temperatures, a distorted version of evaporation of the pendant groups (and end groups) is also possible. As in the case of liquids, where some high energy molecules manage to evaporate, at temperatures well below the boiling point, the same should hold for the case of the pendant groups (or other distinguishable segments) of polymers. Inevitably, this leads to the conclusion that polymers should constantly degrade at room temperature or even at temperatures below zero (°C). To be more accurate, polymers should (latently) decompose at any temperature higher than the distorted melting point of the pendant groups. Extremely low (but not zero) numbers of pendant groups, even at room temperature, will have energy/mobility high enough to cause thermal decomposition. It seems that by increasing temperature, the sensible thermal decomposition is not initiated but simply accelerated.

To avoid any misconceptions, it should be stressed that the decomposition products of latent decomposition would not necessarily be the groups responsible for the matching of the boiling and softening point. For example, in PS, the mobility, collisions, vibrations, molecular interactions, etc., of the benzene group cause the matching and induce decomposition; however, the breaking/decomposition can occur at various random points of the macromolecule (depending on many factors), and thus the benzene will not necessarily be the only or even the major decomposition product. Furthermore, it is not claimed that the decomposition at low temperatures (distorted evaporation) will be detectable in short time experiments and, in general, there is no reason to take for granted that the latent decomposition will be detectable in all polymers at any temperature. In addition, the above claim of latent decomposition can raise various reasonable questions, and the following two questions are perhaps among the most interesting/important: (a) If latent decomposition of polymers is true, then this would mean that elastomers with a glass-to-rubber transition, e.g., at −50 °C decompose at this low temperature and thus, at room temperature (that is a temperature ~70 °C higher than the softening point) should these polymers not be highly unstable and decompose rapidly? (b) An analogous question can be raised for very common thermoplastics, e.g., PP. If PP decomposes at 165 °C during its “melting”, then why in a typical TGA experiment is the onset (sensible) decomposition temperature detected at much higher temperatures, e.g., at 270–300 °C? The mass loss after melting should not increase rapidly, and thus, should the sensible decomposition not occur at lower temperatures and closer to the melting point?

Insights for these issues, and more precisely the absence of detection of mass loss have already been provided based on the absence of volatility of the initial decomposition products and the LLoD of TGA [14]. However, an additional answer to the above questions can be given, by bearing in mind that molecules (pendant groups in this case) exchange energy through collisions. The above mentioned high and low energy states arise from the random collisions of the pendant groups. In the beginning of softening (that is in the solid state) the distances between chains are small, and thus the collisions between the pendant groups are quite frequent and intense despite the relatively low temperature. After polymer relaxation and softening (at the end of softening) the distances have grown, an increase in the free volume has occurred, and thus, although the pendant groups have increased mobility (due to the continuous increase in temperature) the collisions among them are less often and weaker due to the increased distance and the fact that they are bonded to the backbone from one side. Thus, it seems that latent decomposition reaches a maximum at the softening point and then it becomes lower. If the temperature is increased even more, again the intermolecular distances increase further (expansion of matter) but the mobility of the pendant groups is also further increased. After some point, the increase in the mobility and the vibration width of pendant groups overcompensates the increase in distance/volume. Thus, at high temperatures, collisions among pendant groups become again more frequent and intense (effective) and this leads to increased decomposition, that is, the sensible decomposition. Finally, as mentioned many times in the previous sections, not all the decomposition products are volatile and nor can they all react with the remaining polymer. Such effects and depending on the transition temperature can prevent the occurrence of a considerable reduction in molecular weight (thus various properties, e.g., mechanical properties, may not change severely).

The above can explain why latent decomposition seems to continue to a lower extent after softening and in agreement with experimental observations, where in many cases, the decrease in the heat capacity is visible just after melting, but afterwards it continues to increase with rising temperature (the decomposition after softening becomes lower, thus any exothermic effect is less intense and cannot overcompensate the increase in specific heat capacity due to increase in temperature). Additionally, all of these can provide an explanation of how latent decomposition can occur during softening, that is, at much lower temperatures than the onset temperatures of (sensible) decomposition of polymers that is measured by TGA. Finally, these can provide an explanation for the room temperature stability of polymers with sub-zero softening points: in general, thermal motion is a function of (absolute) temperature and at room temperature the mobility is less intense and the collisions of the pendant groups are weak (the exchanged energy is rather low); as a result, the latent decomposition is extremely low and consequently these polymers appear to be fairly stable. In addition, as already mentioned, the increased mobility of pendant groups seems to be crucial for the origin and extent of the latent decomposition but under no circumstance is this the only factor, and the detectability of latent decomposition in all polymers is a matter to be further studied. It is stressed that the above interpretation for the maximum latent decomposition at the softening point concerns polymers where the softening point and the onset decomposition temperature are not close, e.g., for PP. For the case of PVA, the latent decomposition during softening seems to coincide with the onset (sensible) decomposition temperature.

## 5. Conclusions

DSC, TGA and FTIR was used to study the thermal transition behavior of five common thermoplastic polymers. New aspects in the thermal analysis of polymers were introduced. Some of the DSC signal alterations were proven to arise from a minor decomposition that occurs simultaneously with the thermal transitions of polymers. It was proposed and theoretically supported that the simultaneous softening and decomposition is a general and inherent property of all kinds of polymers. It seems that there is a fundamental qualitative similarity in the thermal transition behavior of all polymers (thermoplastic, thermosetting and biopolymers): They cannot be softened, or in other words, they cannot exhibit a glass transition or a melting point without being decomposed. The only difference in their thermal transition behavior is quantitative and lies on the extent of decomposition. An interesting matching of the thermal transition temperature of polymers and the boiling point of their pendant groups (or other distinguishable parts of the macromolecule) can be recognized in many polymers. Based on this, it was proposed that this simultaneous effect of softening and decomposition arises from a distorted version of the boiling of the pendant (and end) groups, which is related to their increased mobility. However, the hidden (latent) decomposition may occur constantly at any temperature higher than the distorted melting point of pendant groups and arises from a distorted version of evaporation of the pendant groups. By increasing temperature, the decomposition of the polymer is not initiated but simply accelerated. Based on all the above, the purely physical nature of the thermal transitions of polymers is disputed.

## Figures and Tables

**Table 1:** % apparent decomposition of various polymers calculated theoretically and % decomposition experimentally observed in the first TGA heating.

	*E_CHEMICAL_* kJ/mol	*E_PHYSICAL_* kJ/mol	*r*	% Apparent Decomposition %*d* = 100/(1 + *r*)	% Decomposition from TGA (1st Heating)
**PP**	610	4	152.5	0.65	<0.08
**PS**	610	4	152.5	0.65	<0.06
**PP-g-MA**	610	4.16	146.6	0.68	0.37 ± 0.1
**PVC**	397	4	99.3	1.00	0.4 ± 0.2
**PVA**	610	25	24.4	3.94	1–1.5
**cellulose**	610	75	8.1	10.95	not applicable
**thermosetting 1**	610	300	2.0	32.97	not applicable
**thermosetting 2**	610	609.9	1.0	50.00	not applicable

## Data Availability

Data are available upon request.

## Supplementary Materials

The following supporting information can be downloaded at: https://www.mdpi.com/article/10.3390/polym14235054/s1, Figure S1: Raw and corrected data of the first scan of the PP sample; Figure S2: Raw and corrected data of Indium measurement; Figure S3: All DSC measurements were performed with pans for this particular batch; Figure S4: Fitting of the DSC curves of PP: (**a**) first scan (**b**) second scan and (**c**) third scan; Table S1: Specific heat of fusion and melting temperature of various Indium measurements.
