# Supplementary material for: Thermochemical Transition in Non-Hydrogen-Bonded Polymers and Theory of Latent Decomposition
Source: Polymers (Basel). 2022 Nov 22;14(23):5054. doi: 10.3390/polym14235054 (PMC9737646; doi:10.3390/polym14235054)
Supplement: Supplementary file 1 [file polymers-14-05054-s001.zip › polymers-2034380-supplementary.pdf]

Supporting information for

Thermochemical transition in non-hydrogen bonded polymers and theory of latent decomposition

Costas Tsioptsias

Laboratory of Physical Chemistry, Department of Chemical Engineering, Aristotle University of Thessaloniki, University Campus, 54124 Thessaloniki, Greece.

Correspondence to: ktsiopts@auth.gr, ktsiopts@gmail.com (C. Tsioptsias).

1.Information for DSC measurements

1.1Manipulation of DSC data

The measurement in the particular DSC instrument can start only if the temperature is below 40 °C. Since sometimes the measurement was started from 20°C and in other case at 30 or 37 °C etc., the high slope (always observed in conventional DSC instruments) of the DSC signal at the beginning of each measurement becomes stable at a different temperature every time. For this reason, the first data points below 50 °C or below 75 °C (depending on how fast stability was reached) have been deleted. For all DSC raw data, correction was performed by subtraction of baseline (empty pan measurement) in order to take into account instrument drift. In the following figures, representative DSC curves for Indium and poly(propylene) (PP) before and after the correction are presented. The dashed line in Figure S1 is the extrapolation of the heat capacity line that is defined from the values <115 °C, by assuming constant mass and linear increase of the specific heat capacity with temperature. The arrow in Figure S1 points out the decrease in heat capacity that is revealed just after "melting" and is realized from the difference between the hypothetical (dashed) line and the actual (solid red) baseline.

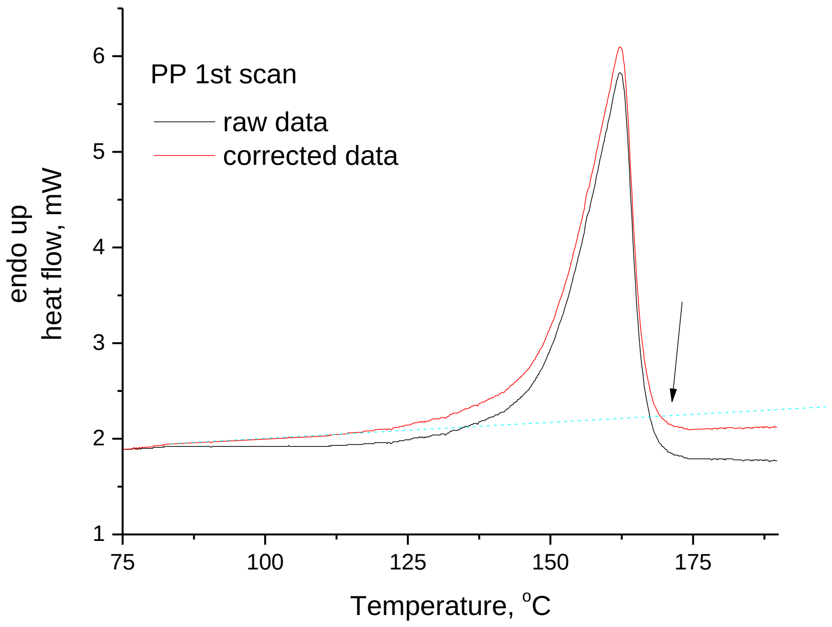

Figure S1. Raw and corrected data of the first scan of the PP sample.

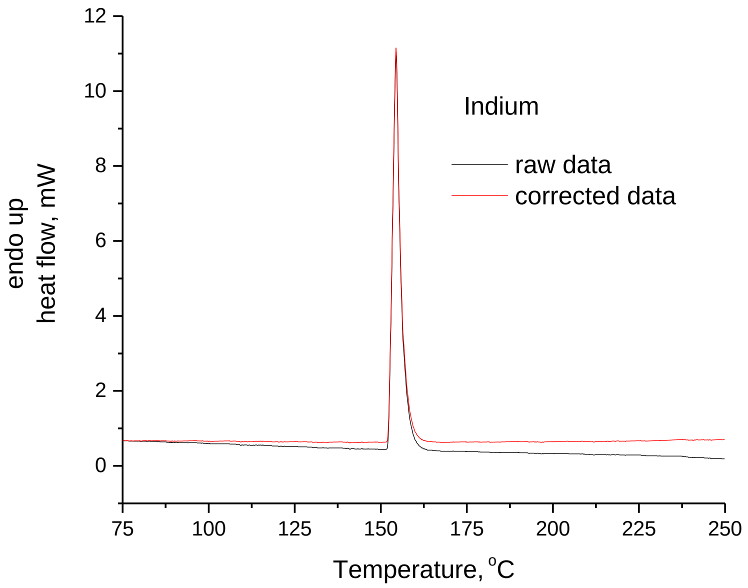

Figure S2. Raw and corrected data of Indium measurement.

1.2 Repeatability of DSC measurements

In Table S1 the specific heat of fusion and the melting temperature of Indium measured in different days and for two different samples are presented. The first two measurements concern an Indium sample with mass 4.7 mg. The pan used for this sample was from an old batch. It was decided that all measurements would better to be performed on new pans of the same batch e.g. to minimize drifting due to minor alterations in the mass of the pans of different batch. Thus, new empty pans (one used as reference pan and one measured as sample to take into account the instrument drift) and a new Indium standard sample was prepared with the new pans of the batch presented in Figure S3. All DSC measurements were performed using pans of this certain batch. This standard Indium sample (mass 6.9 mg) was measured several times at the same days with the measurements of the samples.

Table S1. Specific heat of fusion and melting temperature of various Indium measurements.

**Indium measurement***	**Specific heat of fusion, J/g+**	**Melting temperature, ^oC**	**Mass of sample, mg**
1st	28.45	153.98	4.7
2nd	28.03	153.89	4.7
3rd	28.01	154.49	6.9
4th	27.88	154.40	6.9
5th	28.00	154.44	6.9
6th	28.16	154.44	6.9
7th	28.06	154.41	6.9

*: 1st and 2nd measurements were performed with pans from different batch than all other measurements.

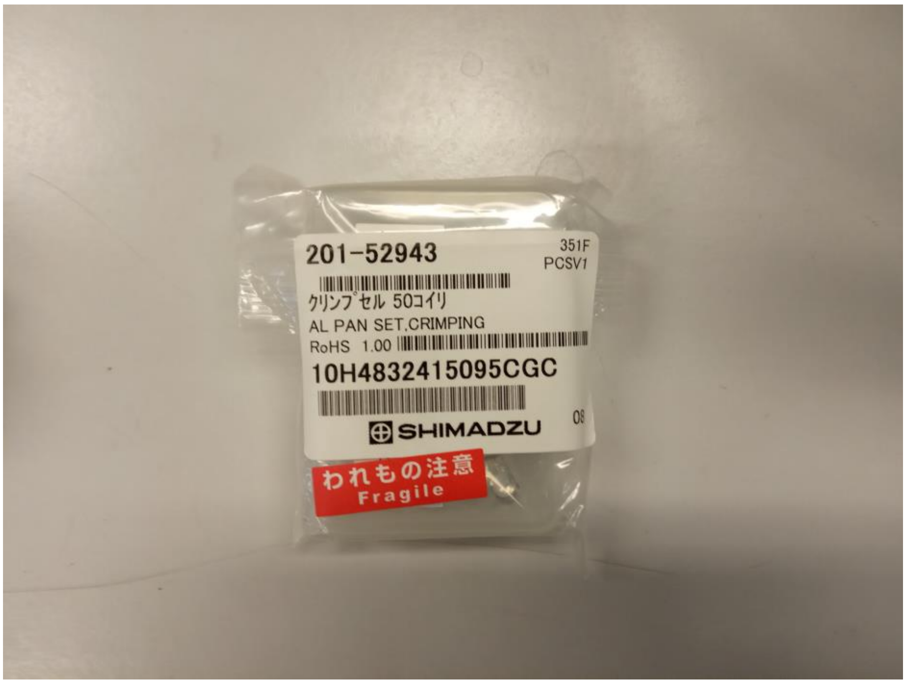

Figure S3. All DSC measurements were performed with pans for this particular batch.

The average value ± standard deviation of all 7 measurements is 28.08 ± 0.18 J/g for specific heat of fusion and 154.29 ± 0.25 ^{o}C for the melting temperature. By comparing the average values with the literature [1] values (28.59 J/g and 156.62 ^{o}C) the deviation is 1.8% for the specific heat of fusion and 1.5% for the melting temperature. Thus, the accuracy of the measurements can be considered to be acceptable.

The average value ± standard deviation of the 5 measurements (3rd-7th) of the same sample with the new pans, is 28.02 ± 0.10 J/g (±0.36%) for specific heat of fusion and 154.44 ± 0.04 ^{o}C for the melting temperature. These standard deviations (of the measurements of the same sample) are indicative of the repeatability of the DSC measurements, which can be considered as fairly good.

2.Information for TGA measurements

The TGA data of the samples were manipulated as follows:

1) The data for temperatures lower than 50 ^{o}C were deleted.

2) The empty pan measurement was subtracted from the samples raw data in order to obtain the corrected data.

3. Other information for the DSC and TGA measurements

For DSC and TGA the maximum heating temperature was based on the thermal transition temperature of each polymer and more specifically they were heated to temperatures about 20 ^oC above their thermal transition temperature. This was chosen in order to keep the applied thermal stress in minimum while ensuring that the thermal transition has ended and baseline is reached. For the case of the PVC sample the end of the thermal transition is around 180 ^oC and thus according to the above concept is should be heated up to 200 ^oC in the DSC. However, from the TGA measurement it is apparent that the (sensible) thermal decomposition for PVC initiates to occur at 200 ^oC. Since for the purpose of this study, the decomposition during or just after softening is of interest and not the sensible decomposition, in the DSC of PVC the lower possible temperature was chosen (180 ^oC) to avoid interference from effects related to the sensible decomposition, e.g. production of increased vapor phase that in the next scan may alter the signal.

For the DSC only one standard (Indium) was used for calibration. Indium exhibits a melting point of 156.6 ^oC and for the four out of five studied polymers (the exception is PS) the temperatures of interest are close to this temperature. For PS only the temperature of the thermal transition is of interest (for the purpose of this study) which is widely known from numerous literature data and thus the correctness for the thermal transition temperature can be readily verified.

4. Additional information for the FTIR measurements

The heating of the polymers, for the FTIR measurements, was performed in situ in the KBr pellets. This approach was adopted in order to be sure that after 3 heating cycles the measurement will be performed at the same spot. FTIR is density/volume dependent. Decomposition is expected to induce density changes due to alterations of the chemical structure.

The polymers were kept in the desired temperature (170 or 130 ^{o}C) for 5 min for the following reasons: 1) The temperature was measured inside the oven and 10 cm away from the glass reactor, 2) The oven has no fan and thus a rapid heat transfer is not likely to occur, 3) There was not preheating of the nitrogen gas that constantly flowed (~20 ml/min, measured by a bubble flowmeter) into and out of the glass reactor. For these reasons it is questionable if the polymers inside the KBr pellets reached the desired temperature. In any case, the discussions from DSC and TGA concern not an exact temperature value but temperature ranges and even if the exact desired temperature of the polymers was not reached even after the 5 min period, the derived conclusions are not at all affected since this means that any of the detected alterations, occurred at even lower temperatures.

For PP, PP-g-MA and PS the decomposition is very low. Thus for these polymers it was chosen to heat them to temperatures slightly above their thermal transition temperatures, that is 130 ^{o}C for PS and 170 ^{o}C for PP and PP-g-MA, in order to increase the possibility of detecting alterations by FTIR. For the case of PVC, at temperatures higher than 200 ^{o}C the decomposition is high, obvious and indisputable and thus it was chosen to study it at lower temperatures. For PVC, the temperature of 170 ^{o}C, which coincides with the end of the thermal transition was considered to be suitable.

The adopted approach (spectra subtraction) is a well-established approach for studying chemical changes in sample and allows for an immediate detection of minor differences in the chemical structure, after each heating, by the appearance of negative and positive peaks. In-situ FTIR measurements have, in general, indisputable advantages over ex-situ measurements like the ones carried out in this study. However, for the phenomena under study, the in situ (at high temperature) measurement would have a great difficulty: the spectra alteration due to alteration of density. On the contrary, the ex-situ measurement in the same spot of the cooled sample limits the interference of spectra alteration due to density alterations. Measurement of the vapor phase either by in-situ FTIR or GS-MS etc. has one “drawback”: By studying the vapor phase only the volatile decomposition products can be detected thus the large (non-volatile) fragments formed at the early stage of decomposition (at lower temperatures) cannot be realized. In other words, by studying the vapor phase, decomposition will be realized, with delay, at higher temperatures.

5. Fitting of the DSC curves of PP

In Figure S4 the fitting of the DSC curves of the three DSC scans of PP are presented.

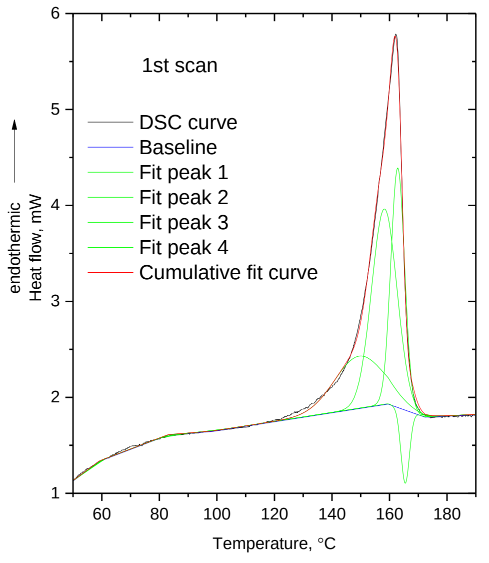

a)

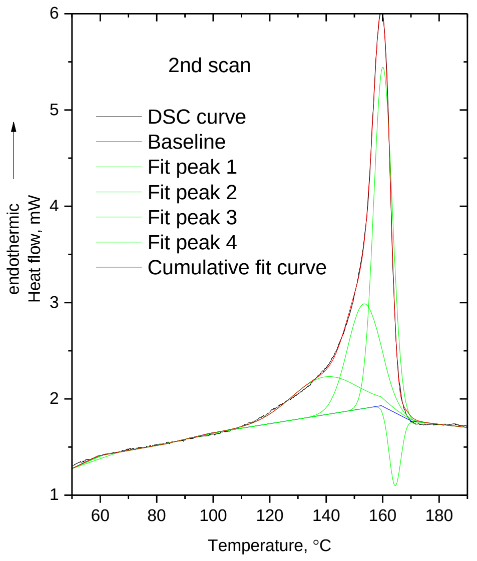

b)

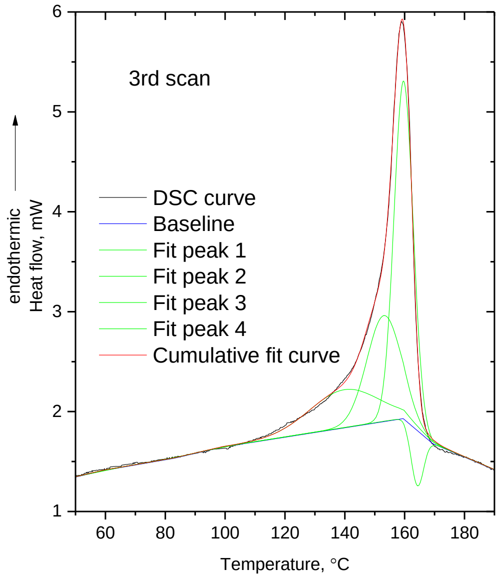

c)

Figure S4. Fitting of the DSC curves of PP: a) first scan b) second scan and c) third scan.
